# Supplementary material for: Alcohol- and Low-Iron Induced Changes in Antioxidant and Energy Metabolism Associated with Protein Lys Acetylation
Source: Int J Mol Sci. 2024 Jul 30;25(15):8344. doi: 10.3390/ijms25158344 (PMC11312970; doi:10.3390/ijms25158344)

SUPPLEMENTAL FIGURES

Figure S1. Western blot analyses of FerL (A), TfR1 (B), and FPN1 (C) proteins and corresponding Ponceau S-stained nitrocellulose membranes were used for normalization of protein loading. Dietary groups are referred to in the text by acronyms control NI (Cont, Normal Iron), EtOH NI (EtOH, Normal Iron), control LI (Cont, Low Iron), EtOH LI (EtOH, Low Iron). Approximately 5 (FerL gel), 10 µg (TfR1 gel), and 15 µg (FPN1) of protein lysates from animal groups were separated on 12% SDS-PAGE and blotted to nitrocellulose membranes. Mean hepatic FerL (Fig. 2C), TfR1 (Fig. 2D), and FPN1 (Fig. 2E) respectively, in whole cell liver lysates presented in the manuscript.

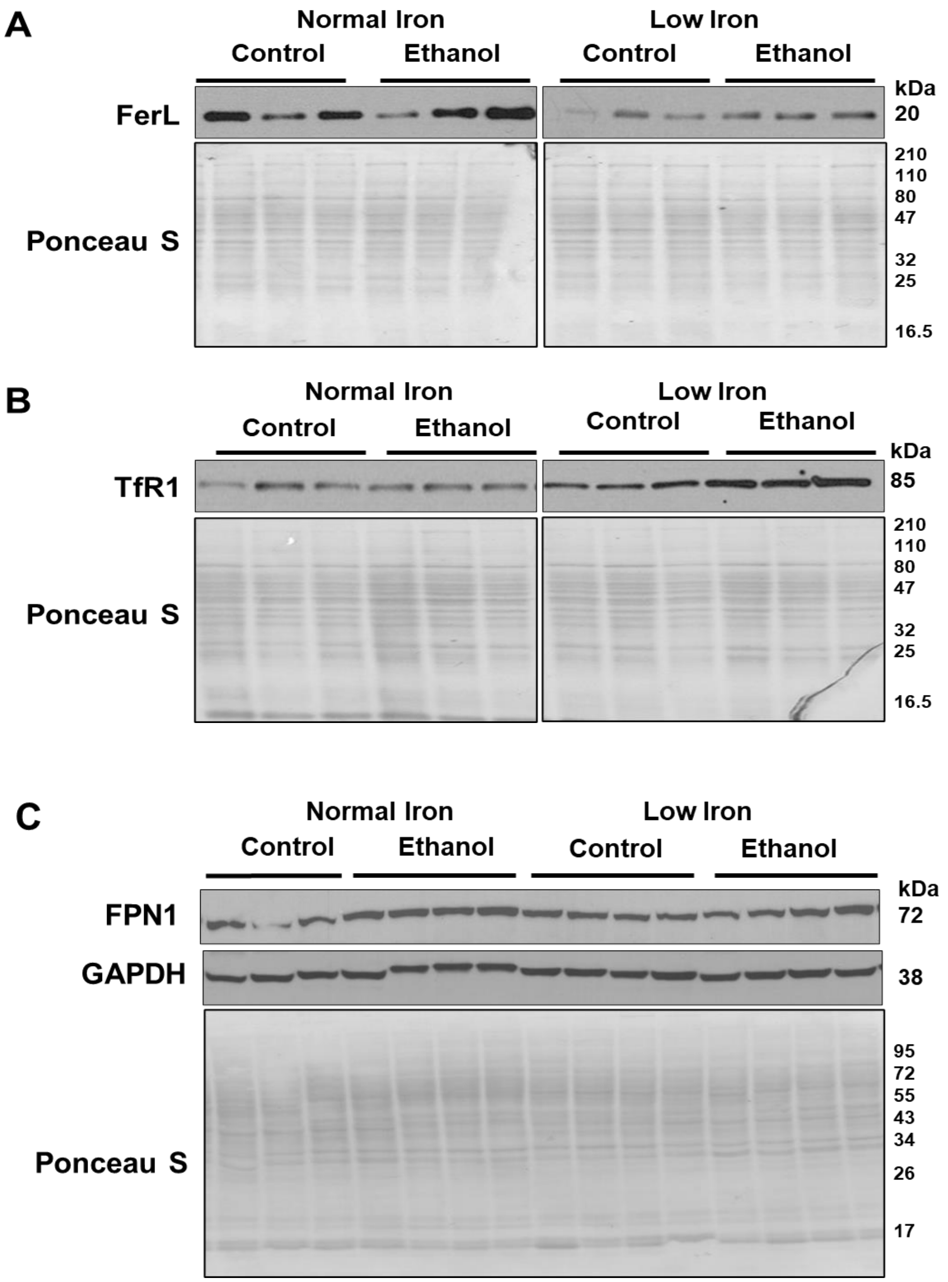

Figure S2. A) Mean total hepatic NAD^+ and NADH (n = 6-8 animals assessed per group). B) Mean oxidized and reduced hepatic glutathione (n = 6-8 animals assessed per group).

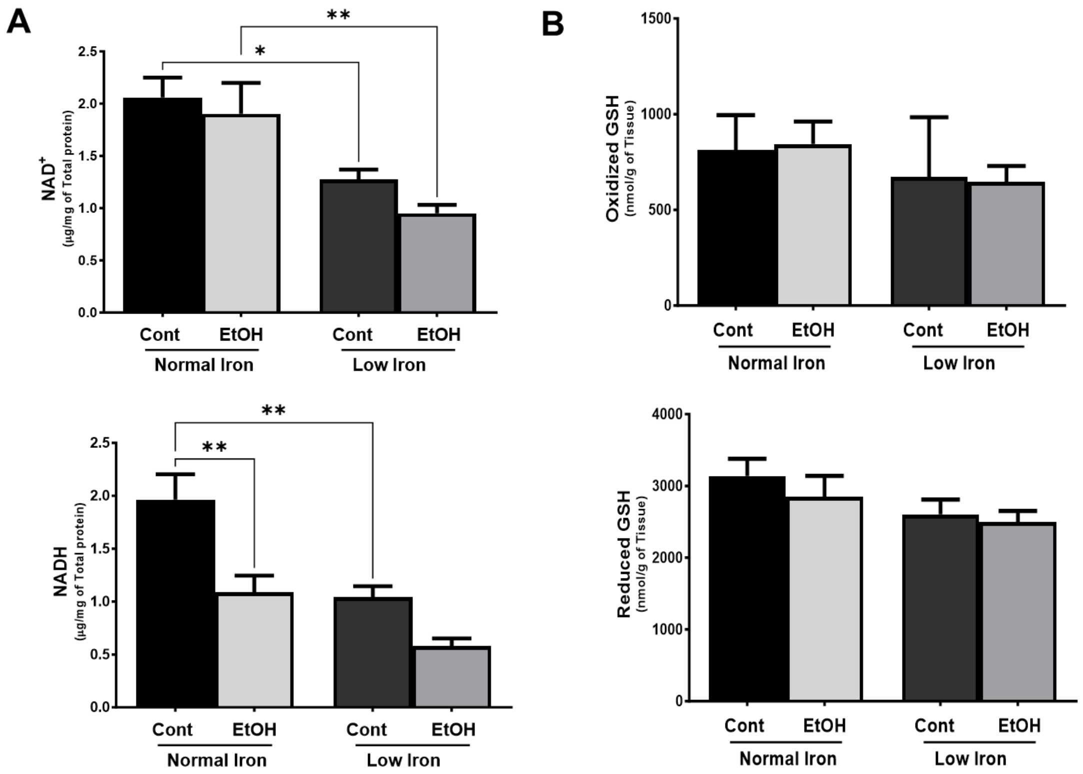

Figure S3. A) Total protein lysine ε-amino acetylation (N-Ac-K) was detected using pan N-acetyl Lys antibody; **(B-D)** SIRT1-3 were detected by corresponding antibodies and Ponceau S-stained nitrocellulose membranes used for normalization of protein loading. Dietary groups are referred to in the text by acronyms control NI (Cont, Normal Iron), EtOH NI (EtOH, Normal Iron), control LI (Cont, Low Iron), and EtOH LI (EtOH, Low Iron). Approximately 30 (N-acetyl Lys gel) and 15 µg (SIRT1 gel) of protein lysates from animal groups were separated on 12% SDS-PAGE and blotted to nitrocellulose membranes. Mean hepatic N-Ac-K (Fig. 4A), SIRT1 (Fig. 4B), SIRT2 (Fig. 4C), and SIRT3 (Fig. 4D), respectively, in liver lysates (n = 7-8 animals assessed per group) presented in the manuscript.

A

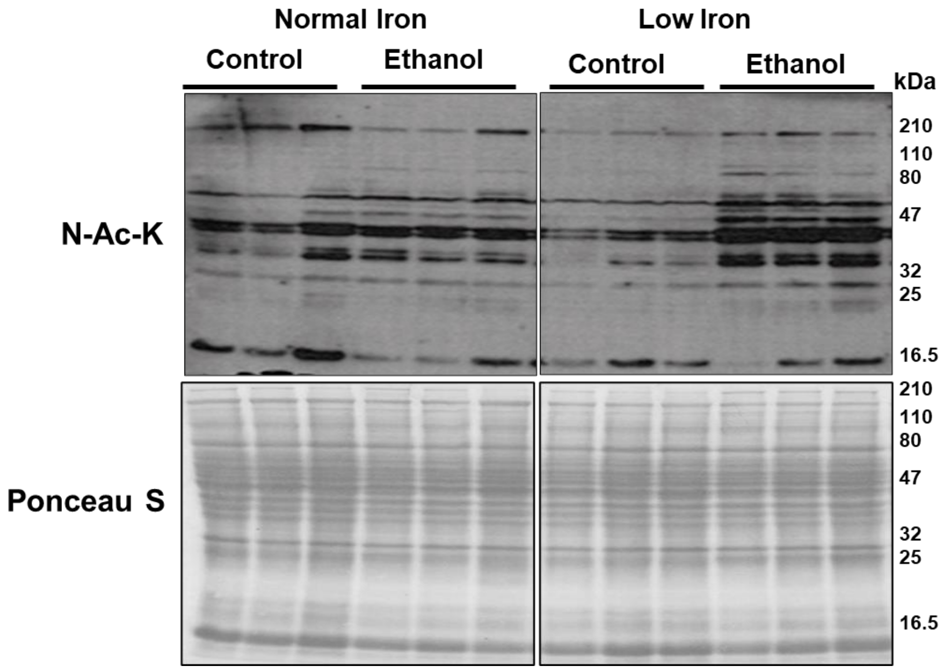

B

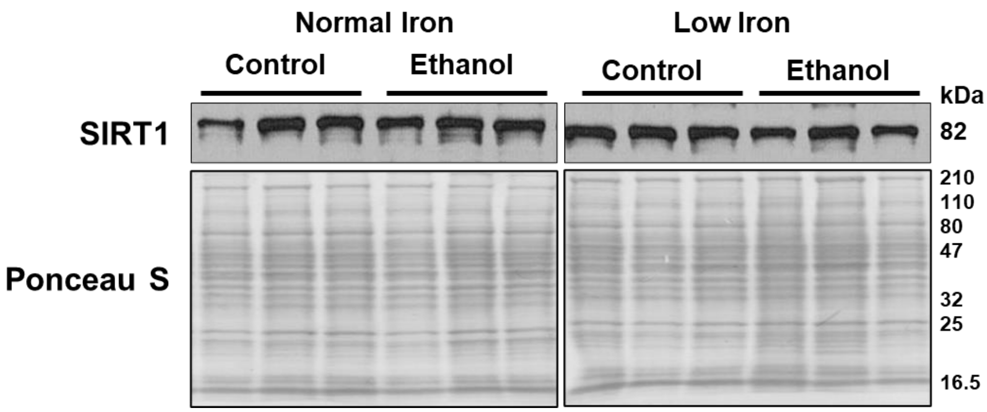

C

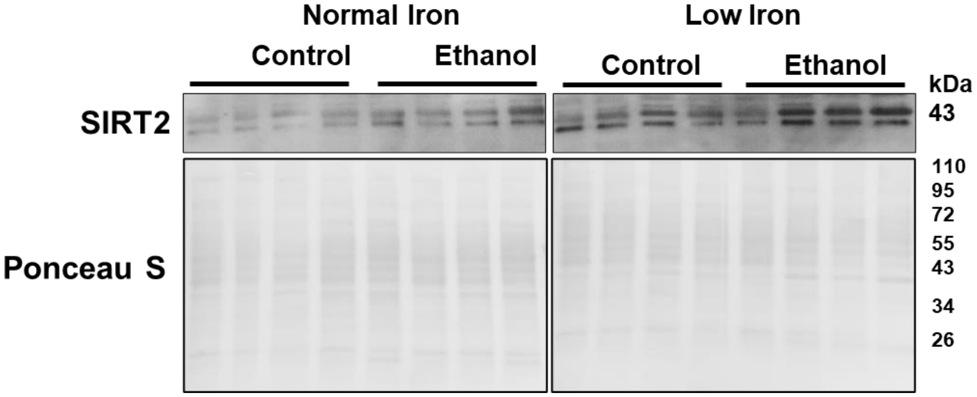
Normal Iron
Low Iron
Control
Ethanol
Control
Ethanol
kDa
SIRT2
43
Ponceau S
110
95
72
55
43
34
26

D

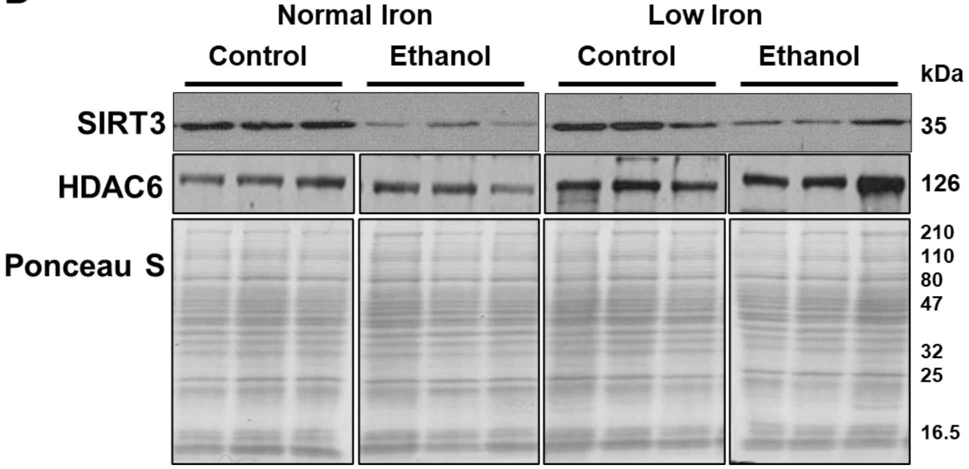
Normal Iron
Low Iron
Control
Ethanol
Control
Ethanol
kDa
SIRT3
35
HDAC6
126
Ponceau S
210
110
80
47
32
25
16.5

Figure S4. **A)** Western blot analyses of SOD1 and GAPDH **(B)** SOD2 Lys acetylation at position 68 (Ac-K68), total SOD2, and GAPDH were detected by corresponding antibodies, and the Ponceau S-stained nitrocellulose membranes used for normalization of protein loading were presented. GAPDH and SOD2 detections were also used to normalize SOD1 and SOD2 Ac-K68 signals. Dietary groups are referred to in the text by acronyms control NI (Cont, Normal Iron), EtOH NI (EtOH, Normal Iron), control LI (Cont, Low Iron), and EtOH LI (EtOH, Low Iron). Approximately 20 µg of protein lysates from each animal group were separated on 12% SDS-PAGE and blotted to nitrocellulose membranes. Mean hepatic SOD1 (Fig. 5A) and SOD2 Ac-K68 (Fig. 5B), were presented in the manuscript.

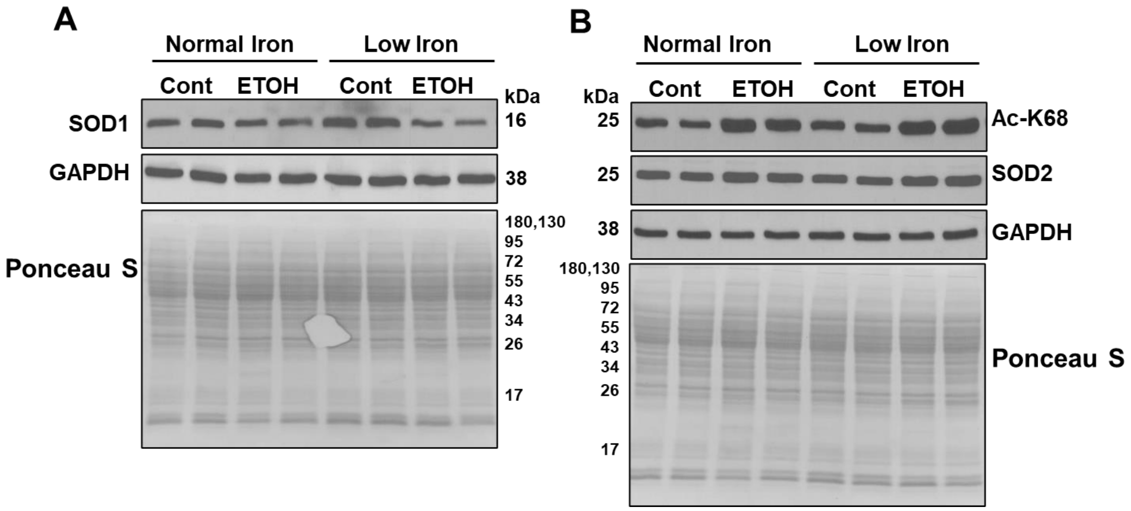

Figure S5. Western blot analyses of OXPHOS subunit expression. Complex II (CII, SDHA, complex V (CV, ATP5A1), complex III (CIII, UCQCRC2), and complex II (CII, SDHB) were detected by corresponding antibodies, and the Ponceau S-stained nitrocellulose membranes used for normalization of protein loading were presented. Dietary groups are referred to in the text by acronyms control NI (Cont, Normal Iron), EtOH NI (EtOH, Normal Iron), control LI (Cont, Low Iron), and EtOH LI (EtOH, Low Iron). Approximately 20 µg of protein lysates from each animal group were separated on 12% SDS-PAGE and blotted to nitrocellulose membranes. Mean hepatic OXPHOS subunit expression (Fig. 6A) was presented in the manuscript.

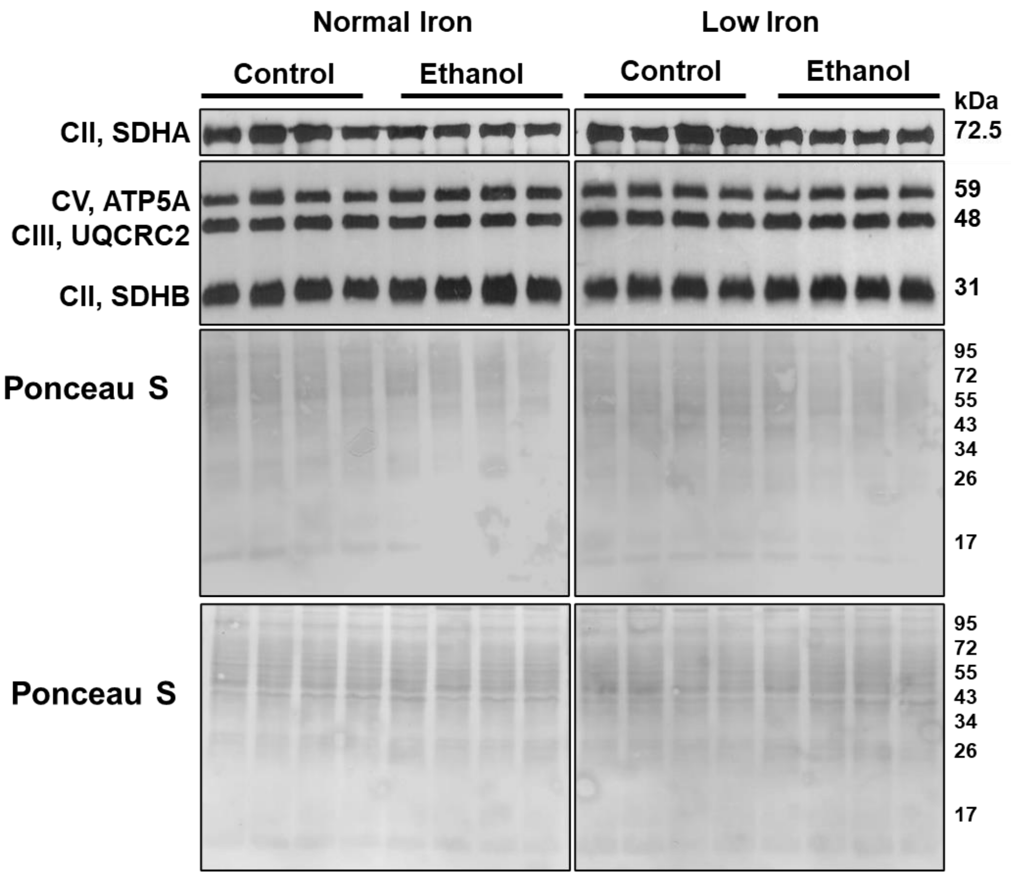

Supplement: Supplementary file 1 [file ijms-25-08344-s001.zip › ijms-3069728-supplementary.pdf]
